# Antimicrobial Resistance: Is the World UNprepared?

**DOI:** 10.1371/journal.pmed.1002130

**Published:** 2016-09-12

**Authors:** The PLOS Medicine Editors

**Affiliations:** The *PLOS Medicine* Editors are Clare Garvey, Thomas McBride, Linda Nevin, Larry Peiperl, Amy Ross, Paul Simpson, and Richard Turner

## Abstract

Long Blurb: On September 21st 2016 the United Nations General Assembly convenes in New York, United States to tackle a looming and seemingly inevitable global challenge with the potential to threaten the health and wellbeing of all people: antimicrobial resistance. In an Editorial, the PLOS Medicine Editors reflect on the challenge of coordinating the response to antimicrobial resistance in order to ensure the viability of current antimicrobials and the development of new therapies against resistant pathogens. Short Blurb: In this month's Editorial, the PLOS Medicine Editors reflect on the upcoming United Nations General Assembly meeting which convenes to discuss the global challenge of antimicrobial resistance.

Some health crises appear suddenly and require swift assessments and strong responses; others come into focus more slowly and are striking in their complexity and intractability. The United Nations (UN) has previously convened high-level meetings and special sessions to focus specifically on health issues on only three occasions: to address the threats posed by HIV, noncommunicable diseases, and Ebola. On September 21st, the UN General Assembly convenes in New York, United States, for a fourth time to tackle a looming and seemingly inevitable global challenge with the potential to threaten the health and wellbeing of all people: antimicrobial resistance (AMR) [1].

The discovery and development of antimicrobial therapies has had a transformational impact on human health over the past century. In high-income countries, few people do not benefit at some point in their lives from the protection afforded by this sizeable battery of drugs against a vast range of fast-evolving pathogenic microorganisms. Yet, despite increasing consumption of antibiotics worldwide, there remains a substantial access problem [2]; for example, around 20% of neonatal deaths result from infections [3]. At the same time, continually emerging resistance to therapies has the potential to undermine the perceived security of the antimicrobial era. A recent review commissioned by the United Kingdom government (The O’Neill Report) estimated that deaths because of antimicrobial resistance could rise from approximately 700,000 deaths a year to close to 10 million deaths per year by 2050 [4].

In an essay published in *PLOS Medicine* earlier this year, Ramanan Laxminarayan and Ranjit Chaudhury highlighted how in India, the world’s largest consumer of antibiotics, a constellation of drivers have created ideal conditions for a rapid rise in resistant infections [5]. These include poor public health infrastructure, a high burden of disease, widespread antibiotic use in animal farming, and the unregulated sale of cheap antibiotics [5]. The drivers and burden of AMR are not unique to India, and they do not respect national borders, GDP, sector, or discipline. Therefore, a coordinated and comprehensive multinational response to address AMR is critical.

September’s meeting is intended to motivate and signal engagement by UN Member States’ highest authorities. Participants will be tackling a One Health issue, meaning that veterinary medicine, agriculture, and environment sectors will play key roles. The sectors that require coordination go beyond the remit of WHO alone, and there have been strong calls for a UN High-Level Coordinating Mechanism on Antimicrobial Resistance to emerge from the meeting [6]. Such a body, perhaps modelled on UNAIDS, would need to have clearly defined resources, goals, and metrics so that all countries can be held accountable for progress against AMR [7].

In writing this editorial, we reached out to a number of prominent researchers, and a universal theme in their opinions was the need to ensure access to antimicrobials for those in need but also to conserve any new therapies to protect against the future development of resistance. Among the many challenges is a pressing need to stimulate research and development on antimicrobial drugs that are effective against multiply resistant pathogens. Weaknesses in the prevailing drug development ecosystem are apparent from the fact that there have been no new classes of antibiotics identified since 1984 [8].

For John-Arne Røttingen, Executive Director of Infection Control and Environmental Health at the Norwegian Institute of Public Health and a coauthor of a recent Chatham House report on antimicrobial resistance, developing a system to “reward innovations that are medically needed through new business models where we pay for the development of innovations and not only for their use in the market place” is one of the biggest priorities. Patrick Vallance, President of Research and Development (R&D) at GlaxoSmithKline, noted “The O’Neill report has suggested several business models and it is important that these get looked at and evaluated. We must ensure that alongside ongoing efforts to address the innovation gap we create mechanisms that decouple financial returns with usage, removing any incentive for unnecessary use of new antibiotics. Pre-purchase agreements, vaccine-like models and others approaches have been proposed.”

Creating a sustainable business model is perhaps the most challenging of the five strategic objectives set out in WHO’s global action plan on antimicrobial resistance [9]. Not only are substantial resources required to advance pathophysiological insights, maintain epidemiological surveillance, and prevent infections while optimizing the use of antimicrobial agents, but a new economic model is also needed to encourage the development of new antimicrobials that is not based on sales volumes.

A model that delinks the cost of R&D from high-volume sales following antibiotic approval has risen to the fore among some advocates because, under such a model, there is the potential to respond to clinical need while ensuring global access and conservation through a series of incentives. A lack of clarity around how to fund such a system of incentives [10] has led to calls for a global fund for R&D against AMR and other infectious public health threats [11].

In the face of such concerns, substantial new initiatives have begun to address the development of new antibiotics. At the beginning of this year, WHO—in partnership with the Drugs for Neglected Diseases initiative (DNDi)—acquired seed funding to launch Global Antibiotic Research & Development (GARD), an organization that aims to apply the principles learned from DNDi’s work in developing tools to combat neglected diseases [12]. Another major new initiative, Combating Antibiotic-Resistant Bacteria Biopharmaceutical Accelerator (CARB-X), based at Boston University, was launched in July to accelerate preclinical development of antibiotics using US$250 million from the Biomedical Advanced Research and Development Authority (BARDA) in the US and matching funds from the United Kingdom’s Wellcome Trust and AMR Centre [8].

September’s high-level meeting at the UN cannot be expected to solve the AMR crisis at a stroke, and the involvement of virtually all countries will be sure to bring together different—and in some cases conflicting—viewpoints and priorities. However, the gathering should take the opportunity to put in place a powerful coordinating body to implement the necessary multifaceted response to AMR [13]. It will be the responsibility of countries and multilateral donors to ensure that adequate financial support is mandated, along with a commitment to create a viable and sustainable model for research and development. Active leadership will be needed to negotiate prudent and explicit country-level standards for antibiotic management, animal husbandry, and other relevant issues. That inaction or lack of coordination could bring the era of effective antibiotic treatment to an end is simply unthinkable.

## Abbreviations

AMR: antimicrobial resistance
BARDA: Biomedical Advanced Research and Development Authority
CARB-X: Combating Antibiotic-Resistant Bacteria Biopharmaceutical Accelerator
DNDi: Drugs for Neglected Diseases initiative
GARD: Global Antibiotic Research & Development
R&D: research and development
UN: United Nations
